# Delays and disparities in access to secure psychiatric services in England: national study of referrals and access assessments

**DOI:** 10.1192/bjo.2026.12053

**Published:** 2026-07-24

**Authors:** Sarah-Jayne Leonard, Jana Bowden, Florian Walter, Jon Fernando Carey, Louise Robinson, Caroline Logan, Ruth McDonald, Jennifer Shaw, Jane Senior

**Affiliations:** Division of Psychology and Mental Health, University of Manchester, UK; Division of Nursing and Midwifery, University of Manchester, UK; Social Sciences, Northumbria University, UK; Department of Security and Crime Science, University College London, UK; Population Health Sciences Institute, Newcastle University, UK; Lancashire and South Cumbria NHS Foundation Trust, Preston, UK

**Keywords:** Prison, secure psychiatric services, care pathways, assessment, access to care

## Abstract

**Background:**

Secure psychiatric services (SPS) are vital for people with severe mental disorders who pose significant risks, yet national evidence on referral pathways, acceptance and timeliness of admission is limited. Delays and variability in access are especially concerning in prisons, where mental health crises are frequent and alternatives limited.

**Aims:**

This study presents the first national analysis of SPS referral and admission patterns in England following the introduction of provider collaboratives. It identifies predictors of referral acceptance and delays across the referral-to-admission pathway to inform equity and performance improvement.

**Method:**

Data were drawn from 149 low-secure and 98 medium-secure services across 15 provider collaboratives, encompassing 1356 referrals. Multivariable models examined predictors of acceptance and delay, benchmarked against national policy standards.

**Results:**

Almost half of referrals originated from prisons (46%, *n* = 529), with 55% accepted. Acceptance was associated with gender, legal status, Mental Health Act status, prior secure admission, diagnosis, referral origin and reason for referral, but not with ethnicity or urgency. Substantial site-level variation reflected local practice and resources. Delays were widespread: fewer than a third of urgent referrals were assessed within 2 days, and only 19–25% of accepted prison referrals met recommended assessment and admission targets. Although prison referrals were processed more quickly than community or SPS referrals, national standards were seldom achieved.

**Conclusions:**

Access to SPS remains inconsistent, with substantial variation in acceptance and widespread delays across referral pathways. Despite faster processing of prison referrals, national assessment and admission standards were rarely met, particularly for this group. These findings highlight gaps between policy expectations and practice, indicating a need for improved adherence to national timeframes, greater standardisation of decision-making and enhanced system oversight, to reduce inequities in access.

Secure psychiatric services (SPS; often referred to internationally as forensic mental health services) are a critical component of mental health provision at the intersection of health, social care and the Criminal Justice System in England and Wales. They provide specialist mental healthcare for individuals whose mental disorders are associated with a significant risk of harm to others, delivered across low, medium and high levels of security to ensure the least restrictive environment compatible with safety and therapeutic need.^1,2^ SPS primarily serve individuals with severe and enduring mental illnesses, including psychotic disorders, personality disorders and neurodevelopmental conditions. Many individuals have a history of serious offending linked to their mental illness, whereas others present with complex clinical and risk profiles that cannot be managed safely within other settings. The dual mandate of SPS is to address mental health needs while ensuring public protection through safe and effective risk management.^3^ However, access to these services is constrained by limited capacity, variable admission thresholds and complex referral pathways, raising concerns about equity, timeliness of admission and adherence to the principle of least restrictive care. These challenges are particularly acute for individuals referred from prison settings, where delays in transfer to appropriate care may exacerbate clinical risk and raise ethical and human rights concerns.

## Access to low- and medium-SPS

Determining eligibility for admission to low- and medium-SPS is complex^4^ and contemporary evidence is limited. Earlier studies found wide variation in admission criteria across services, shaped by contextual factors (e.g. regional need and service configuration)^5^ and individual considerations (e.g. diagnosis, offence characteristics and availability of alternative pathways^6^). This variability limits the generalisability of single-site studies. National-level evidence remains scarce, with only one comprehensive study, conducted two decades ago, examining assessment and admission to medium-SPS.^7^ Based on 418 assessments across 34 services, it identified discrepancies between assessed need and admission: around 20% of those deemed to require medium security were not admitted, whereas some admitted individuals were assessed as requiring lower or no security. The authors concluded that demand exceeds capacity and that limited provision leads to inappropriate use of medium-secure beds, contravening the principle of least restrictive care under the Mental Health Act 1983. Since then, the independent sector has become a major provider of SPS in England, now delivering approximately 32% of provision. This shift from supplementary to core provision is likely to affect admission pathways, thresholds and patient flow, but its impact remains underexplored.

Referrals to SPS originate from the community, in-patient services and the criminal justice system. Community referrals arise when escalating risk cannot be safely managed in less secure settings, whereas in-patient referrals occur when clinical complexity or risk exceeds the remit of general psychiatric services or requires movement between levels of security. Within the criminal justice pathway, referrals may arise from prisons (e.g. acute mental illness in custody), courts (e.g. fitness to plead or psychiatric disposals) or police custody (e.g. need for urgent assessment). Historically, transfers from prison to SPS in England and Wales have been particularly challenging. The Bradley Review^8^ set a 14-day target for transfer of prisoners with acute severe mental illness; although some progress was reported, delays and regional variation persisted.^9^ In 2021, National Health Service (NHS) England introduced guidance specifying a maximum of 14 days from referral to assessment, and a further 14 days to admission for accepted cases.^10^ Despite this, inspection reports from His Majesty’s Inspectorate of Prisons continue to highlight transfer delays.^11^ System-level constraints persist, including assessment bottlenecks, limited bed capacity and regional variation, with implications for patient care, legal compliance and human rights. The Mental Health Act 2025 has now formalised this timeframe into a statutory 28-day limit.

## Provider collaboratives

NHS England has recently devolved the commissioning of adult low- and medium-SPS to regional provider collaboratives, NHS-led partnerships of NHS Trusts and independent sector providers,^12,13^ whereas high-SPS continue to be directly commissioned at a national level by NHS England. Initially piloted under the ‘New Care Models’ programme, 15 adult secure provider collaboratives became operational across England between July 2021 and May 2022. The aim is to improve patient pathways, enhance cross-sector collaboration and ensure a more responsive, needs-led approach to SPS. A key element of this model is the Access Assessment Service (AAS), a single point of referral into secure care. Referrals for admission to low- and medium-secure care, regardless of originating service, are submitted to the AAS with an indicated level of security, alongside clinical and risk information as determined by each AAS. The AAS is designed to streamline assessments by reducing duplication, delay and variation. Access assessments are complex and require specialist clinical formulation of both mental health needs and risk. NHS England’s National Service Specifications set standards for timely, robust decision-making with (a) urgent referrals: assessment within a maximum of 2 days from receipt, with the outcome determined within 24 h of the assessment; and (b) routine referrals: assessment within 21 days of receipt. These standards also require the consistent application of the principle of least restrictive care.^1,2^

There is currently limited evidence on how reliably AASs are being implemented across regions, including whether services meet required timeframes. This study addresses this gap by providing the first national evaluation of referrals to low- and medium-SPS in England following the introduction of provider collaboratives. Specifically, it examines (a) the predictors of referral acceptance and (b) factors associated with delays in assessment and admission.

## Method

### Design

A retrospective audit was conducted of all referrals to 149 low- and 98 medium-SPS across all 15 provider collaborative sites over a 6-month period.

### Ethical standards

Ethical approval was received from the University of Manchester Research Ethics Committee (proportionate panel) on 20 July 2022 (UREC ref. no. 2022-14938-24544). The study was conducted as a national service evaluation, with NHS England acting as the data controller and request approver, given its commissioning responsibility for all low- and medium-SPS. NHS England formally requested data from each provider collaborative site for the specified timeframes. The clinical lead at each provider collaborative site reviewed the request and agreed to participate, with all sites providing data in response to this national request. Data-sharing was conducted in line with NHS England governance processes, and only anonymised, routinely collected service data were provided to the research team.

### Sample and timeframe

Data were collected for all referrals to the 15 provider collaboratives, over a 6-month period, for assessment for admission to approximately 6000 beds. These beds are distributed across 247 discretely commissioned services (60.3% low-SPS, 39.7% medium-SPS; 25.5% female SPS, 74.5% male SPS).

Not all provider collaboratives became operational on the same date. Therefore, to ensure consistency across the data, provider collaboratives were asked to provide data from the first day of the fourth month following the provider collaborative ‘go-live’ date. For example, if a provider collaborative go-live date was 1 June 2021, data were requested for the timeframe of 1 October 2021 to 31 March 2022. In most cases, this involved requesting data from the single point of access; however, at three provider collaborative sites, data requests were required for each trust/provider individually.

### Data

We requested anonymised, routinely collected data variables for all people referred for access assessment during the study period. The requested variables are presented Table 1, along with the number of provider collaborative sites that returned the data variables and the proportion of patients for whom the variables are available across the full data-set. The least frequently reported variables were primary diagnoses (*n* = 8 provider collaborative sites) and date of assessment (*n* = 9 provider collaborative sites). Variables with the most missing data were Mental Health Act status (43.1%) and whether the referral was outside of the patient’s ‘natural clinical flow’ (42.6%^a^).

**Table 1:** Data availability for all variables Table 1 long description.

Variables	Sites returned	Referrals, *N* = 1356	
		*n* (%)	
		Data available	Data missing
Demographics			
Gender	15 (100)	1323 (97.6)	33 (2.4)
Ethnicity	12 (80.0)	1041 (76.8)	315 (23.2)
Criminal status	13 (86.7)	997 (73.5)	359 (26.5)
Mental Health Act status	13 (86.7)	772 (56.9)	584 (43.1)
Primary diagnosis	8 (53.3)	687 (50.7)	667 (49.3)
Referral			
Date of referral	15 (100)	1354 (99.9)	2 (0.1)
Type of referral	14 (93.3)	1165 (85.9)	191 (14.1)
Outside of natural clinical flow^a^	10 (66.7)	779 (57.4)	577 (42.6)
Referring service	14 (93.3)	1211 (89.3)	145 (10.7)
Referrer identified security level	13 (86.7)	1028 (75.8)	328 (24.2)
Primary reason for referral	12 (80.0)	1009 (74.4)	341 (25.6)
Assessment			
Date of assessment	9 (60.0)	1016 (74.9)	340 (25.1)
Assessing service security level	13 (86.7)	960 (70.8)	396 (29.2)
Date of referral outcome	13 (86.7)	1180 (87.0)	176 (13.0)
Referral outcome	15 (100)	1319 (97.3)	37 (2.7)
Admission			
Date of admission	13 (86.7)	587 (92.7)	46 (7.3)
Admission destination: security level	13 (86.7)	523 (82.6)	110 (17.4)

a. Patients referred to out-of-area secure psychiatric services.

### Procedure

The clinical leads at each provider collaborative were contacted to nominate a colleague who was best placed to assist with data return. This professional was then provided with the data request instructions and a bespoke Microsoft Excel data capture file. Data were returned to the research team via encrypted email.

### Analysis

Descriptive data (frequencies and averages) were computed using SPSS (version 24). To examine variation in referral outcomes and timelines, we conducted multivariable regression analyses. Referral outcomes (accepted versus declined) were modelled using mixed-effects logistic regression. Referral timelines were analysed as continuous outcomes, including time from referral to admission, time from assessment to admission and time from assessment outcome to admission, using linear mixed-effects models. All models included a random intercept for site, to account for clustering by referring organisation. Fixed effects included gender, ethnicity, criminal justice status, Mental Health Act status, previous secure admission, primary diagnosis, referral source, type of referral (urgent versus routine) and reason for referral. Predictor significance was evaluated using Wald χ^2^ statistics. Results are reported as odds ratios with 95% confidence intervals for binary outcomes, and adjusted mean differences with confidence intervals for continuous outcomes. For continuous outcomes, group differences were estimated using linear contrasts of model coefficients derived from the mixed-effects models. Models were estimated on a complete-case basis, with observations with missing data excluded from relevant analyses. Multiple imputation was not undertaken because missingness was largely structural rather than item-level, with some variables not returned by all sites rather than being sporadically missing (e.g. primary diagnosis was returned by 8 of 15 sites). Imputation of variables would require unverifiable assumptions about the missing data mechanisms, and would risk the creation of inappropriate precision. All analyses were conducted using Stata (version 15).

## Results

The total number of referrals across provider collaborative sites was 1356. A descriptive overview is provided below for available data, which are presented in Tables 2–4.

**Table 2:** Descriptive data for patient referrals

Variables	*n* (%)
Demographics	
Gender (*n* = 1323, 97.6%)	
Male	1121 (84.7)
Female	202 (15.3)
Ethnicity (*n* = 1041, 76.8%)	
Asian/Asian British	94 (9.0)
Black/Black British/Caribbean/African	184 (17.7)
Mixed or multiple ethnic groups	49 (4.7)
White	619 (59.5)
Other	95 (9.1)
Criminal status (*n* = 997, 73.5%)	
Sentenced	391 (39.2)
Convicted and awaiting sentence	21 (2.1)
On remand and awaiting trial	216 (21.7)
Civil	369 (37.0)
Mental Health Act status (*n* = 772, 56.9%)^a^	
s. 37 hospital order	62 (8.0)
s. 37/41 hospital order with restrictions	159 (20.6)
s. 38 interim hospital order	8 (1.0)
s. 45A hospital admission (‘hybrid’ order)	5 (0.6)
s. 48/49 removal to hospital of other prisoners (with restriction)	112 (14.5)
s. 47/49 removal to hospital of sentenced prisoners (with restriction)	103 (13.3)
s. 2 admission for assessment	24 (3.1)
s. 3 admission for assessment and treatment	265 (34.3)
Informal	29 (3.8)
Suppressed^b^	5 (0.6)
Second or subsequent secure psychiatric services admission (*n* = 748, 55.2%)	
Yes	362 (48.4)
Primary diagnosis (*n* = 687, 50.7%)	
Schizophrenia or other primary psychotic disorders^c^	482 (70.2)
Personality disorder and related traits^d^	75 (10.9)
Bipolar or related disorders^e^	19 (2.8)
Depressive disorders	10 (1.5)
Disorders of intellectual development	38 (5.5)
Autism spectrum disorder	32 (4.7)
Acquired brain injury	13 (1.9)
Other^f^	18 (2.6)
Outside of natural clinical flow (*n* = 779, 57.4%)	
Yes	176 (22.6)
Type of referral (*n* = 1165, 85.9%)	
Routine^g^	866 (74.3)
Urgent^h^	299 (25.7)
Referring service (*n* = 1211, 89.3%)	
Prison	558 (46.1)
Police/courts	19 (1.6)
Low-secure	94 (7.8)
Medium-secure	148 (12.2)
High-secure	45 (3.7)
PICU	174 (14.4)
Locked rehabilitation	13 (1.1)
General acute	107 (8.8)
Primary care (GP, other)	40 (3.3)
Other^f^	13 (1.1)
Level of security identified as required by the referrer (*n* = 1028, 75.8%)	
Low	462 (44.9)
Medium	555 (54.0)
Female blended services	11 (1.1)
Primary reason for referral (*n* = 1009, 74.4%)	
Step-up to higher security	33 (3.3)
Step-down to lower security	113 (11.2)
Movement from CJS to secure services	516 (51.1)
Movement from the community	45 (4.5)
Movement to a different service at the same security level	68 (6.7)
Movement from non-secure^i^	234 (23.2)
Assessing service security level (*n* = 960, 70.8%)	
Low	409 (42.6)
Medium	551 (57.4)
Referral outcome (*n* = 1319, 97.3%)	
Accepted	633 (48.0)
Declined	621 (47.1)
Withdrawn	53 (4.0)
Redirected	12 (0.9)
Admission destination security level (*n* = 523, 82.6%^j^)	
Low	235 (44.9)
Medium	288 (55.1)

PICU, psychiatric intensive care unit; GP, general practitioner; CJS, Criminal Justice System.

a. At time of referral, or if detained in prison, were the person to be admitted.

b. Including CP(I)A, s.36 and s.42.

c. Including schizophrenia, schizoaffective disorder, psychosis and delusional disorder.

d. Including antisocial, borderline, dissocial, emotionally unstable, organic, avoidant and schizotypal personality disorders.

e. Including bipolar affective disorder and hypomania.

f. Descriptors omitted due to counts being less than 5.

g. Defined by the presence of immediate and unmanageable risk requiring rapid assessment.

h. Defined as the presence of significant need for secure care but allowing for assessment within standard timeframes.

i. Including PICU and general acute.

j. Of those admitted.

**Table 3:** Multilevel logistic regression analysis examining demographic, clinical and referral-related predictors of assessment outcomes across sites Table 3 long description.

Variables	Wald χ^2^	Wald *p*-value	Odds ratio (95% CI)	Site variance (95% CI)
Gender (female)	*χ* ^2^(1) = 8.84	0.003	0.593 (0.42–0.84)	0.233 (0.10–0.54)
Ethnicity (overall)	*χ* ^2^(4) = 4.72	0.317	–	0.189 (0.07–0.48)
Asian/Asian British	–	Reference	–	–
Black/Black British/Caribbean/African	–	0.226	0.716 (0.42–1.23)	–
Mixed or multiple ethnic groups	–	0.182	0.599 (0.28–1.27)	–
White	–	0.067	0.635 (0.39–1.03)	–
Other	–	0.049	0.531 (0.28–0.997)	–
Criminal status (overall)	*χ* ^2^(3) = 21.86	<0.001	–	0.415 (0.14–1.22)
Sentenced	–	Reference	–	–
Convicted and awaiting sentence	–	0.006	17.02(2.22−130.47)	–
On remand and awaiting trial	–	0.021	1.55(1.07−2.25)	–
Not subject to criminal proceedings	–	0.077	0.75(0.55−1.03)	–
Mental Health Act status (overall)	*χ* ^2^(4) = 36.53	<0.001	–	0.524 (0.19–1.46)
Informal	–	Reference	–	–
Civil	–	0.006	3.99 (1.50–10.66)	–
Hospital order	–	<0.001	7.26 (2.70–19.53)	–
Prison transfer – sentenced	–	<0.001	8.09 (2.83–23.16)	–
Prison transfer – remand	–	<0.001	12.81 (4.44–36.97)	–
Previous secure admission	*χ* ^2^(1) = 4.82	0.0282	1.42 (1.04–1.95)	0.168 (0.05–0.53)
Primary diagnosis group (overall)	*χ* ^2^(3) = 10.35	0.0158	–	–
Personality disorder	–	Reference	–	–
Severe mental illness	–	0.003	2.23 (1.31–3.77)	–
Neurological	–	0.232	1.51 (0.77–2.96)	–
Other	–	0.229	1.74 (0.71–4.29)	–
Origin of referral (overall)	*χ* ^2^(3) = 23.63	<0.001	–	0.327 (0.13–0.83)
Prison	–	Reference	–	–
Secure services	–	0.439	1.14 (0.82–1.58)	–
In-patient (non-secure)	–	<0.001	0.50 (0.37–0.69)	–
Other	–	0.733	0.91 (0.53–1.56)	–
Type of referral (urgent)	*χ* ^2^(1) = 1.89	0.170	1.27 (0.90–1.78)	0.115 (0.04–0.33)
Reason for referral (overall)	*χ* ^2^(5) = 17.94	0.003	–	0.234 (0.09–0.59)
Step-up to higher security	–	Reference	–	–
Step-down to lower security	–	0.191	1.77 (0.75–4.14)	–
Movement from CJS to secure services	–	0.145	1.79 (0.82–3.94)	–
Movement from community to secure services	–	0.583	1.32 (0.48–3.67)	–
Movement to a different service but at the same security level	–	0.512	1.36 (0.54–3.40)	–
Movement from non-secure	–	0.775	0.89 (0.39–2.01)	–

Reference, baseline category against which all other subcategories within each predictor are compared; Wald χ^2^ statistics, *p*-values and odds ratios are not applicable to reference categories. CJS, Criminal Justice System.

**Table 4:** Linear mixed-effects regression results for factors associated with time intervals from referral, assessment and outcome to admission

Variables	Referral to admission (coefficient [95% CI], *p*)	Assessment to admission (coefficient [95% CI], *p*)	Outcome to admission (coefficient [95% CI], *p*)
Criminal status	Wald *χ* ^2^ = 1.51, *p* = 0.6800	Wald *χ* ^2^ = 2.71, *p* = 0.4381	Wald *χ* ^2^ = 2.05, *p* = 0.5623
Sentenced	Reference	Reference	Reference
Convicted and awaiting sentence	−8.79 [−53.87, 36.30], *p* = 0.702	−22.32 [−61.98, 17.35], *p* = 0.270	−17.50 [−60.40, 25.40], *p* = 0.424
On remand and awaiting trial	−10.78 [−31.12, 9.56], *p* = 0.299	−11.65 [−29.61, 6.32], *p* = 0.204	−10.51 [−30.45, 9.44], *p* = 0.302
Not subject to criminal proceedings	−10.17 [−29.07, 8.73], *p* = 0.292	−10.89 [−27.94, 6.16], *p* = 0.211	−11.64 [−29.97, 6.70], *p* = 0.213
Mental Health Act status	Wald *χ* ^2^ = 3.79, *p* = 0.4349	Wald *χ* ^2^ = 4.55, *p* = 0.3365	Wald *χ* ^2^ = 5.05, *p* = 0.2821
Informal	Reference	Reference	Reference
Civil	−3.91 [−16.68, 8.87], *p* = 0.550	−4.57 [−17.29, 8.15], *p* = 0.476	−4.82 [−17.72, 8.09], *p* = 0.463
Hospital order	−8.00 [−22.27, 6.27], *p* = 0.266	−9.86 [−24.69, 4.97], *p* = 0.193	−9.25 [−23.93, 5.43], *p* = 0.215
Prison transfer – sentenced	−9.39 [−25.52, 6.73], *p* = 0.258	−9.78 [−25.58, 6.02], *p* = 0.227	−10.47 [−26.17, 5.23], *p* = 0.193
Prison transfer – remand	−12.77 [−31.27, 5.72], *p* = 0.175	−13.65 [−32.42, 5.13], *p* = 0.153	−14.19 [−32.87, 4.48], *p* = 0.138
Previous secure admission	Wald *χ* ^2^ = 2.58, *p* = 0.1082	Wald *χ* ^2^ = 5.57, *p* = 0.0182	Wald *χ* ^2^ = 7.57, *p* = 0.0059
No	Reference	Reference	Reference
Yes	9.93 [0.13, 19.73], *p* = 0.0478	13.58 [2.36, 24.80], *p* = 0.0182	3.71 [−12.29, 19.72], *p* = 0.641
Primary diagnosis group	Wald *χ* ^2^ = 0.58, *p* = 0.9003	Wald *χ* ^2^ = 0.55, *p* = 0.9084	Wald *χ* ^2^ = 1.37, *p* = 0.7116
Personality disorder	Reference	Reference	Reference
Severe mental illness	−3.97 [−15.33, 7.38], *p* = 0.489	−6.56 [−18.18, 5.07], *p* = 0.270	−5.37 [−17.34, 6.61], *p* = 0.373
Neurological	−3.27 [−16.51, 9.98], *p* = 0.624	−4.99 [−18.36, 8.38], *p* = 0.462	−3.97 [−18.14, 10.20], *p* = 0.585
Other	−5.27 [−20.52, 9.98], *p* = 0.491	−6.21 [−21.57, 9.15], *p* = 0.419	−4.51 [−20.28, 11.25], *p* = 0.572
Referral service	Wald *χ* ^2^ = 54.36, *p* ≤ 0.001	Wald *χ* ^2^ = 31.12, *p* ≤ 0.001	Wald *χ* ^2^ = 48.86, *p* ≤ 0.001
Prison	Reference	Reference	Reference
Secure services	60.38 [43.04, 77.71], *p* ≤ 0.001	50.12 [31.67, 68.57], *p* ≤ 0.001	57.95 [40.86, 75.03], *p* ≤ 0.001
In-patient (non-secure)	−9.91 [−22.94, 3.11], *p* = 0.133	−9.97 [−23.46, 3.51], *p* = 0.145	−9.28 [−22.47, 3.91], *p* = 0.164
Other	−6.52 [−19.00, 5.95], *p* = 0.306	−6.57 [−19.84, 6.70], *p* = 0.328	−6.21 [−18.93, 6.51], *p* = 0.337
Type of referral	Wald *χ* ^2^ = 30.54, *p* ≤ 0.001	Wald *χ* ^2^ = 15.48, *p* ≤ 0.001	Wald *χ* ^2^ = 13.43, *p* ≤ 0.001
Routine	Reference	Reference	Reference
Urgent	−50.45 [−68.34, −32.56], *p* ≤ 0.001	−38.35 [−57.45, −19.24], *p* ≤ 0.001	−32.59 [−50.01, −15.16], *p* ≤ 0.001
Reason for referral pathway	Wald *χ* ^2^ = 69.08, *p* ≤ 0.001	Wald *χ* ^2^ = 38.73, *p* ≤ 0.001	Wald *χ* ^2^ = 54.69, *p* ≤ 0.001
Step-up to higher security	Reference	Reference	Reference
Step-down to lower security	−13.83 [−65.24, 37.59], *p* = 0.598	−18.07 [−70.31, 34.17], *p* = 0.498	−14.17 [−63.23, 34.89], *p* = 0.571
Movement from Criminal Justice System to secure services	−85.38 [−134.14, −36.62], *p* = 0.001	−84.89 [−136.03, −33.76], *p* = 0.001	−80.19 [−126.52, −33.86], *p* = 0.001
Movement from community to secure services	−119.27 [−180.58, −57.95], *p* ≤ 0.001	−105.90 [−165.74, −46.07], *p* = 0.001	−99.28 [−158.16, −40.40], *p* = 0.001
Movement to a different service but at the same security level	−8.49 [−63.45, 46.47], *p* = 0.762	−8.49 [−63.45, 46.47], *p* = 0.762	−9.77 [−62.37, 42.82], *p* = 0.716
Movement from non-secure	−97.87 [−148.92, −46.83], *p* ≤ 0.001	−87.21 [−140.27, −34.14], *p* = 0.001	−83.68 [−132.38, −34.98], *p* = 0.001

Reference, baseline category against which all other subcategories within each predictor are compared.

### Demographic and clinical characteristics of the patient sample

Data on gender were available for 97.6% of patients (Table 2), with the majority being male (*n* = 1121, 84.7%). Ethnicity was recorded for 76.8% (*n* = 1041); of these, 59.5% were White (*n* = 619), 17.7% Black/Black British/Caribbean/African (*n* = 184), 9% Asian/Asian British (*n* = 94), 4.7% mixed/multiple ethnic groups (*n* = 49) and 9.1% other ethnicities (*n* = 95). Criminal status was available for 73.5% (*n* = 997), with 63% (*n* = 628) involved in criminal proceedings. Among these, 41.3% were convicted prisoners (*n* = 412) and 21.7% were on remand (*n* = 216). Civil patients constituted 37% (*n* = 637). Mental Health Act status was recorded for 56.9% (*n* = 772), with 58.2% detained under Part 3 (*n* = 449), including 27.8% transferred from prison and 30.2% under hospital orders. A further 37.4% were detained under civil sections and 3.8% (*n* = 29) admitted informally.

Primary diagnosis data were available for 50.7% (*n* = 687), with schizophrenia and related psychotic disorders being the most common (70.2%, *n* = 482). Other diagnoses included personality disorders (10.9%), intellectual disability (5.5%), autism spectrum disorder (4.7%), bipolar disorders (2.8%), acquired brain injury (1.9%), depressive disorders (1.5%) and other (2.6%). Referral type was known for 85.9% (*n* = 1165), with the majority being routine (74.3%, *n* = 866). Referring service was provided for 89.3% (*n* = 1211); most referrals came from prison (46.1%, *n* = 558), followed by secure services (23.7%), psychiatric intensive care units (PICU) (14.4%), general acute services (8.8%) and other sources, including primary care, police/courts and locked rehabilitation.

Referrers identified the level of security required for 75.8% (*n* = 1028), with 54% for medium-secure (*n* = 555), 44.9% for low-secure (*n* = 462) and 1.1% for blended female services (*n* = 11). Assessment of service security level was available for 70.8% (*n* = 960), with 57.4% assessed for medium-secure (*n* = 551) and 42.6% for low-secure (*n* = 409). The primary reason for referral was documented for 74.4% (*n* = 1009), with the most common being diversion from the Criminal Justice System (51.1%, *n* = 516). Other reasons included step-down to lower security (11.2%), lateral moves (6.7%), escalation (3.3%) and referrals from non-secure services (23.2%) and the community (4.5%).

### Referral and assessment information

#### Referral-to-assessment timeframe

Referral-to-assessment time was available for 71.0% of patients (*n* = 963), with a mean duration of 18.2 days (s.d. 22.3, range 0–213). Urgent referrals were assessed within a mean of 8.9 days (s.d. 12.4), with 28.6% seen within 2 days.^1,2^ Routine referrals had a mean assessment time of 18.7 days (s.d. 19.3), with 72.1% assessed within 21 days.^1,2^ The shortest assessment times were for referrals from general acute services (mean 12.8 days, s.d. 11.9), followed by PICU (14.6 days, s.d. 17.8), prison (16.7 days, s.d. 18.6), medium-secure units (MSU; 19.3 days, s.d. 18.0), low-secure units (LSU; 21.1 days, s.d. 28.5) and high-secure units (HSU; 33.5 days, s.d. 41.3). Less than half of prison referrals (41.7%, *n* = 168) did not meet the recommended maximum of 14 days between referral and assessment.^10^

#### Assessment outcome

Assessment outcomes were available for 1319 patients (97.3%), with 633 accepted (48.0%) and 621 declined (47.1%). The remaining patients were either withdrawn by the referrer (*n* = 53, 4%) or redirected to other provider collaboratives (*n* = 12, 0.9%). Assessment outcomes by referral source and primary diagnosis are presented in Table 3.

Female referrals had lower odds of acceptance than male referrals (odds ratio 0.59, 95% CI 0.42–0.84; *p* = 0.003). There was no clear overall effect of ethnicity (*p* = 0.317), although the ‘other’ group showed marginally lower odds (odds ratio 0.53, 95% CI 0.28–0.997; *p* = 0.049). Criminal status strongly predicted acceptance (*p* < 0.001): compared with those sentenced, individuals convicted and awaiting sentence were far more likely to be accepted (odds ratio 17.02, 95% CI 2.22–130.47; *p* = 0.006), as were those on remand (odds ratio 1.55, 95% CI 1.07–2.25; *p* < 0.021). Mental Health Act status showed increasing odds of acceptance from civil detentions (odds ratio 3.99, 95% CI 1.50–10.66; *p* = 0.006) through hospital orders (odds ratio 7.26, 95% CI 2.70–19.53; *p* < 0.001) to prison transfers, both sentenced (odds ratio 8.09, 95% CI 2.83–23.16; *p* < 0.001) and on remand (odds ratio 12.81, 95% CI 4.44–36.97; *p* < 0.001). A prior secure admission also increased odds of acceptance (odds ratio 1.42, 95% CI 1.04–1.95; *p* = 0.028). Primary diagnosis overall was significantly associated with acceptance (*p* = 0.016), but only severe mental illness was more likely to be accepted than personality disorder (odds ratio 2.23, 95% CI 1.31–3.77; *p* = 0.003). Referral source was predictive (*p* < 0.001), driven by in-patient non-secure referrals being less likely to be accepted than prison referrals (odds ratio 0.50, 95% CI 0.37–0.69; *p* < 0.001). Unlike referral reasons (*p* = 0.003), urgency of referral had no significant effect (*p* = 0.170).

The impact of the examined factors on the likelihood of acceptance differed across provider collaborative sites, with the greatest variability seen for Mental Health Act status (variance 0.52, 95% CI 0.19–1.46) and criminal status (variance 0.42, 95% CI 0.14–1.22).

Estimates for smaller subgroups were associated with wide confidence intervals, reflecting limited precision due to small numbers within certain categories, and these should therefore be interpreted with caution. A breakdown of referral outcomes by referral source, including outcomes for non-accepted referrals, is provided in Supplementary Table 1 available at https://doi.org/10.1192/bjo.2026.12053.

#### Referral-to-assessment outcome timeframe

Referral-to-assessment outcome time was available for 1122 patients (82.7%), with an average duration of 31.3 days (s.d. 31.9), ranging from 0 to 278 days. For urgent referrals, the average time from referral to outcome was 17 days (s.d. 21.3). Routine referrals received an outcome on average 33.4 days (s.d. 30.7) following referral. Referrals from general acute services received an outcome the fastest on average (21.6 days, s.d. 22.0), followed by prison (29.8 days, s.d. 30.2), PICU (30 days, s.d. 31.8), LSU (32.2 days, s.d. 32.6) and MSU (33.2 days, s.d. 23.7), with referrals from HSU taking the longest on average (51 days, s.d. 45.6).

#### Assessment-to-assessment outcome timeframe

Assessment-to-assessment outcome time was available for 885 patients (62.3%), with an average duration of 15.7 days (s.d. 23.7), ranging from 0 to 239 days. For urgent referrals, the average time from assessment to outcome was 6.3 days (s.d. 15.1). Routine referrals received an outcome on average 17.8 days (s.d. 23.6) after assessment. Outcomes were provided fastest following assessment for referrals from general acute services (9.1 days, s.d. 16.4), followed by MSU (14.8 days, s.d. 15.8), prison (14.9 days, s.d. 23.4), LSU (15.4 days, s.d. 17.8), PICU (16.2 days, s.d. 26.7) and HSU, which had the longest average duration (25.4 days, s.d. 22.3). For 92 urgent referrals (46.2%), the assessment outcome was provided within 24 h of the assessment.^1,2^

### Admission information

#### Admission destination security level

Admission destination security level was recorded for 523 admitted patients (82.6%). Of these, 288 (55.1%) were admitted to medium-secure care and 235 (44.9%) to low-secure care.

#### Referral-to-admission timeframe

Referral-to-admission time was available for 544 admitted patients (85.9%), with an average duration of 74.3 days (s.d. 82.5), ranging from 0 to 884 days. For urgent referrals, the average time between referral and admission was 38.2 days (s.d. 35.0). Routine referrals were admitted on average 89.2 days (s.d. 92.7) following referral. Referrals from general acute services experienced the shortest average time to admission (40.3 days, s.d. 28.1), followed by PICU (52.2 days, s.d. 61.6), prison (60.5 days, s.d. 49.1), LSU (101.5 days, s.d. 104.8) and MSU (114 days, s.d. 139.4). Referrals from HSU experienced the longest average time to admission (182.2 days, s.d. 90.6). Just under a quarter of referrals from prison (*n* = 64, 24.8%) met the recommended maximum of 28 days from referral to admission, as per good clinical practice guidelines.^10^

In multivariable mixed-effects models (Table 4), referral source and urgency were the only consistent predictors of admission delay. Transfers from other secure services waited substantially longer than prison referrals, whereas urgent referrals were admitted markedly faster than routine ones. Other factors, including criminal status, Mental Health Act status and diagnosis, were not significant once site variation was accounted for.

#### Assessment-to-admission timeframe

Assessment-to-admission time was available for 442 patients admitted (69.8%), with an average duration of 59.6 days (s.d. 76.2), ranging from 0 to 645 days. For urgent referrals, the average time between assessment and admission was 29.5 days (s.d. 29.8). Routine referrals were admitted on average 74 days (s.d. 87.1) following assessment. Referrals from general acute services were admitted the fastest on average (32.7 days, s.d. 27.2), followed by prison (46 days, s.d. 48.1), PICU (46.7 days, s.d. 67.1), MSU (89.2 days, s.d. 119), LSU (109 days, s.d. 124.5) and HSU, which had the longest average time to admission following assessment (160.9 days, s.d. 86.2). Only 18.7% (*n* = 40) of referrals from prison met the good clinical practice recommendation of a maximum of 14 days from psychiatric assessment to admission.^10^

Patterns mirrored those observed for referral-to-admission. Urgent referrals and those from prisons or general hospitals reached admission more rapidly, whereas transfers from secure services experienced significantly longer waits (Table 4). No independent effects were detected for patient-level variables such as criminal status, Mental Health Act status or diagnosis once site variation was accounted for.

#### Assessment outcome-to-admission timeframe

Assessment outcome-to-admission time was available for 493 admitted patients (77.9%), with an average duration of 47 days (s.d. 76.9), ranging from 0 to 814 days. For urgent referrals, the average time from assessment outcome to admission was 25 days (s.d. 28.3). Routine referrals were admitted on average 58.8 days (s.d. 89.1) following assessment outcome. Referrals from general acute services had the shortest average time to admission following assessment outcome (26 days, s.d. 26.4), followed by prison (32.2 days, s.d. 39.7), PICU (33.5 days, s.d. 62.4), LSU (74.2 days, s.d. 104.7), MSU (88.6 days, s.d. 139.5) and HSU, with the longest average time (135.6 days, s.d. 80.2).

Consistent with earlier models, referral context and urgency were the main determinants of delay (Table 3). Transfers from other secure services took several weeks longer than prison referrals, whereas urgent cases progressed to admission sooner. No significant effects were identified for diagnosis, Mental Health Act status or criminal justice status once clustering by site was included.

## Discussion

This study provides a national overview of referrals and admissions to SPS across England, highlighting variation in acceptance rates, timeliness and the influence of referral source and patient characteristics. The findings highlight systemic pressures and possible inequities in access to SPS, with implications for policy, clinical decision-making and service configuration.

### Predictors of acceptance and delays in referral and admission pathways

The study identified 1356 referrals to SPS, the majority originating from prison settings, with almost half of the total accepted for admission (48%). Factors that significantly predicted acceptance included gender, legal status, Mental Health Act status, previous secure admission, diagnosis, referral origin and reason for referral. Urgency of the referral did not influence the likelihood of acceptance. Diagnostic factors may also shape access, because individuals with personality disorder have historically faced barriers to secure psychiatric care due to perceptions of risk, stigma and the legacy of the former ‘treatability’ criterion within the Mental Health Act, which contributed to the prioritisation of those with severe mental illness.^14^ Consistent with this, some secure-based clinicians report that such settings are unsuitable for managing personality disorder, with some noting that a primary diagnosis of personality disorder can lead to refusal of admission in the absence of a secondary diagnosis or dedicated treatment provision.^15^ Considerable variation in admission and acceptance rates was observed across sites, suggesting that local factors such as service configuration, capacity, clinical culture, admission criteria, commissioning priorities, bed management practices and the availability of specialist provisions (e.g. women’s secure beds) may influence referral acceptance and admission thresholds, although these factors were not directly examined in the current study. These findings highlight the need for clearer, more standardised, national admission criteria across services to ensure consistency, fairness and transparency in decision-making. They also suggest that national thresholds for acceptance, particularly for women and for those with non-psychotic disorders, should be reviewed to reduce unwarranted variation and potential inequities in access to SPS. Further exploration is needed to understand the underlying causes of these discrepancies, including whether they reflect variations in throughcare efficiency, service capacity or broader cultural and professional factors influencing admission decisions.

Significant delays were observed across all stages of the referral-to-admission pathway, with provider collaboratives not consistently meeting the timescale requirements set out in the AAS specification^1,2^ and National Prison Transfer and Remission Guidance.^10^ These findings predate recent updates to the Mental Health Act, which have since formalised a statutory 28-day limit from referral to admission for those referred from prison. Against this backdrop, performance remains far from the level required to meet these strengthened legal standards: the recommended maximum timeframe of 28 days was met in only 25% of prison referrals accepted for admission, and just 19% met the standard of admission within 14 days of psychiatric assessment, with the longest wait extending to 467 days. Understanding why these delays occur, and why performance varies across services, is essential to inform strategies that could replicate best practice and improve consistency in the referral-to-admission pathway. Barriers to delivering best practice will be explored in a subsequent paper, presenting a qualitative analysis of clinicians’ experiences of the referral and assessment care pathway nationally. This will also consider how differences across services, such as bed numbers, average length of stay and broader local pathways, may influence both access and timeliness for SPS.

These findings highlight a critical need to review and strengthen pathway processes, with a focus on improving timeliness and ensuring adherence to national standards to promote equitable and effective access to SPS. They also point to a substantial implementation gap, indicating that significant system-level changes will be necessary before the revised Mental Health Act standards can be reliably achieved in practice. There is an urgent need to reduce delays for those in prison, which is neither a place of safety nor equipped to manage individuals in acute psychiatric crisis. Such delays can result in prolonged periods without appropriate treatment, with prisoners awaiting treatment often managed in seclusion, which is highly detrimental to mental health.

This national evaluation of SPS referrals and admissions highlights substantial variability in acceptance rates and critical delays across the national referral-to-admission pathway. Patient characteristics, referral source and site-level factors influenced both access and timeliness, revealing systemic pressures and potential inequities in secure care. Prolonged waits and inconsistent access carry significant clinical and risk implications, because individuals who could benefit from secure care are often managed in less appropriate settings or have their treatment delayed. The findings underscore the need to standardise admission criteria, optimise referral pathways and ensure compliance with national timeframes, particularly for people in prison. Barriers to best practice, including service configuration, resource limitations and inconsistent data recording, must be addressed. Further research is needed to understand variation across provider collaborative sites, and to identify evidence-based models of good practice that can be replicated nationally. Ensuring timely and equitable access to SPS is essential to safeguard patient outcomes and reduce avoidable risk across the system.

### Strengths and limitations

This study represents the first national evaluation of referrals and access assessments to low- and medium-SPS in England since the introduction of provider collaboratives. A major strength is the large, multicentre data-set covering all 15 provider collaboratives and providing a comprehensive picture of contemporary referral pathways. By analysing routinely collected, real-world data, the study captures practice as it occurred within operational systems. The inclusion of both patient- and system-level predictors within multivariable models allows a nuanced understanding of factors influencing referral outcomes and timeliness, and the focus on pathway timeframes directly benchmarks practice against national standards. Nevertheless, data returns varied across provider collaborative sites, with substantial gaps for key variables such as primary diagnosis, Mental Health Act status and date of assessment. Missing data on date of assessment is particularly problematic, because it directly limits the ability to evaluate timeliness of access against national standards and, therefore, to assess service performance. The complete-case approach used for regression analyses may introduce bias if missingness is not random. Given the proportion of missing data for some variables, the analytic sample may differ from the full cohort, potentially affecting the comparability of estimates. This is particularly relevant for variables such as diagnosis and Mental Health Act status, where high levels of missingness mean that observed associations may not fully reflect patterns in the overall referral population.

We do not present descriptive data stratified by gender (e.g. ethnicity within gender), because the small number of female referrals would have resulted in small cells. Inference for categorical predictors relied on the Wald test, which can be less reliable for coefficients of large magnitude;^16,17^ in those cases, it tends to understate rather than overstate statistical significance. This is relevant to only a small number of sparse subgroups with extreme odds ratios in this analysis, not affecting variables central to our conclusions.

The incomplete recording of even basic patient information is a significant concern that undermines clinical governance, service planning and the ability to conduct robust research. It is unclear why these data could not be provided in full, given that such variables would be expected to be routinely recorded in clinical systems. This variability highlights an urgent need for consistent, high-quality data capture across provider collaborative sites, including consideration of mandatory reporting for key performance indicators such as assessment and admission timeframes. Unmeasured influences, such as local service configuration, clinical risk assessment practices and real-time bed availability, are also likely to have shaped decision-making. Although the national scope is a strength, differences in data quality and recording practices between provider collaboratives may have introduced variability not fully accounted for in analyses. Finally, because the study covers the early phases of provider collaborative implementation, findings may not reflect longer-term adaptations in practice.

## Supporting information

10.1192/bjo.2026.12053.sm001 Leonard et al. supplementary material Leonard et al. supplementary material

## Data Availability

The data that support the findings of this study are available from the corresponding author, S.-J.L., upon reasonable request.

## Footnotes

a. Patients who are referred to out-of-area SPS.

## Supplementary material

The supplementary material is available online at https://doi.org/10.1192/bjo.2026.12053

## Table 1 Long description

The table presents data availability for all variables related to referrals, including the number of provider collaborative sites that returned the data variables and the proportion of patients for whom the variables are available across the full data-set. The table has 3 columns: Variables, Sites returned, and Referrals. The Referrals column is further divided into Data available and Data missing. The Variables column lists different categories such as Demographics, Referral, Assessment, and Admission. Each row provides specific details under these categories. For example, under Demographics, it lists Gender, Ethnicity, Criminal status, Mental Health Act status, and Primary diagnosis. The Sites returned column indicates the number of sites that returned data for each variable, and the Referrals column shows the number and percentage of data available and data missing for each variable. Notable trends include the least frequently reported variables being primary diagnoses with data from 8 provider collaborative sites and date of assessment with data from 9 provider collaborative sites. Variables with the most missing data are Mental Health Act status with 43.1% missing and whether the referral was outside of the patient’s natural clinical flow with 42.6% missing.

Navigate back to Table 1.

## Table 3 Long description

The table presents a multilevel logistic regression analysis examining demographic, clinical, and referral-related predictors of assessment outcomes across sites. It includes six columns: Variables, Wald chi-square, Wald p-value, Odds ratio (95% CI), and Site variance (95% CI). The table has 25 rows, including row labels and values. Row 1: Gender (female), 2(1), 8.84, 0.003, 0.593 (0.42-0.84), 0.233 (0.10-0.54). Row 2: Ethnicity (overall), 2(4), 4.72, 0.317, 0.189 (0.07-0.48). Row 3: Asian/Asian British, Reference, Reference. Row 4: Black/Black British/Caribbean/African, 0.226, 0.716 (0.42-1.23). Row 5: Mixed or multiple ethnic groups, 0.182, 0.599 (0.28-1.27). Row 6: White, 0.067, 0.635 (0.39-1.03). Row 7: Other, 0.049, 0.531 (0.28-0.997). Row 8: Criminal status (overall), 2(3), 21.86, <0.001, 0.415 (0.14-1.22). Row 9: Sentenced, Reference, Reference. Row 10: Convicted and awaiting sentence, 0.006, 17.02 (2.22-130.47). Row 11: On remand and awaiting trial, 0.021, 1.55 (1.07-2.25). Row 12: Not subject to criminal proceedings, 0.077, 0.75 (0.55-1.03). Row 13: Mental Health Act status (overall), 2(4), 36.53, <0.001, 0.524 (0.19-1.46). Row 14: Informal, Reference, Reference. Row 15: Civil, 0.006, 3.99 (1.50-10.66). Row 16: Hospital order, <0.001, 7.26 (2.70-19.53). Row 17: Prison transfer sentenced, <0.001, 8.09 (2.83-23.16). Row 18: Prison transfer remand, <0.001, 12.81 (4.44-36.97). Row 19: Previous secure admission, 2(1), 4.82, 0.028, 1.42 (1.04-1.95), 0.168 (0.05-0.53). Row 20: Primary diagnosis group (overall), 2(3), 10.35, 0.0158. Row 21: Personality disorder, Reference, Reference. Row 22: Severe mental illness, 0.003, 2.23 (1.31-3.77). Row 23: Neurological, 0.232, 1.51 (0.77-2.96). Row 24: Other, 0.229, 1.74 (0.71-4.29). Row 25: Origin of referral (overall), 2(3), 23.63, <0.001, 0.327 (0.13-0.83). Row 26: Prison, Reference, Reference. Row 27: Secure services, 0.439, 1.14 (0.82-1.58). Row 28: In-patient (non-secure), <0.001, 0.50 (0.37-0.69). Row 29: Other, 0.733, 0.91 (0.53-1.56). Row 30: Type of referral (urgent), 2(1), 1.89, 0.170, 1.27 (0.90-1.78), 0.115 (0.04-0.33). Row 31: Reason for referral (overall), 2(5), 17.94, 0.003, 0.234 (0.09-0.59). Row 32: Step-up to higher security, Reference, Reference. Row 33: Step-down to lower security, 0.191, 1.77 (0.75-4.14). Row 34: Movement from CJS to secure services, 0.145, 1.79 (0.82-3.94). Row 35: Movement from community to secure services, 0.583, 1.32 (0.48-3.67). Row 36: Movement to a different service but at the same security level, 0.512, 1.36 (0.54-3.40). Row 37: Movement from non-secure, 0.775, 0.89 (0.39-2.01).

Navigate back to Table 3.
