# Supplementary material for: Delays and disparities in access to secure psychiatric services in England: national study of referrals and access assessments
Source: BJPsych Open. 2026 Jul 24;12(4):e198. doi: 10.1192/bjo.2026.12053 (PMC13419692; doi:10.1192/bjo.2026.12053)
Supplement: Leonard et al. supplementary material [file S2056472426120535sup001.docx]

| **Referral source** | **Accepted** | **Declined** | **Withdrawn** | **Redirected** | **Total (n)** |
| --- | --- | --- | --- | --- | --- |
| Prison | 53.38 | 43.33 | 2.01 | 1.28 | 547 |
| Secure services | 56.74 | 37.94 | 4.61 | 0.71 | 282 |
| Inpatient (non-secure) | 36.84 | 57.19 | 4.91 | 1.05 | 285 |
| Other | 45.83 | 47.22 | 6.94 | 0.00 | 72 |
| **Total** | 49.75 | 45.62 | 3.63 | 1.01 | 1,186 |

Supplementary table: Outcomes of referrals by referral source (%)

Percentages are based on all referrals with a recorded outcome (accepted, declined, withdrawn, or redirected). The acceptance rate cited in the abstract for prison referrals (55%) is calculated on accepted and declined cases only, excluding withdrawn and redirected referrals.
